# Elephant Endotheliotropic Herpesvirus 4 and *Clostridium perfringens* Type C Fatal Co-Infection in an Adult Asian Elephant (*Elephas maximus*)

**DOI:** 10.3390/ani12030349

**Published:** 2022-01-31

**Authors:** Taiana Costa, Guido Rocchigiani, Flavia Zendri, Gabby Drake, Javier Lopez, Julian Chantrey, Emanuele Ricci

**Affiliations:** 1 Department of Veterinary Anatomy, Physiology and Pathology, Institute of Infection, Veterinary and Ecological Sciences, Leahurst Campus, University of Liverpool, Chester High Road, Neston CH64 7TE, UK; taiana.costa@liverpool.ac.uk (T.C.); g.rocchigiani@liverpool.ac.uk (G.R.); flavia.zendri@liverpool.ac.uk (F.Z.); chantrey@liverpool.ac.uk (J.C.); 2 Chester Zoo, Upton-by-Chester, Cheshire CH2 1LH, UK; g.drake@chesterzoo.org (G.D.); j.lopez@chesterzoo.org (J.L.)

**Keywords:** *Clostridium* spp., *Clostridium perfringens* type C, clostridial enterotoxemia, elephant endotheliotropic herpesvirus, EEHV, elephant endotheliotropic herpesvirus hemorrhagic disease, *Elephas maximus*

## Abstract

**Simple Summary:**

Elephant endotheliotropic herpesvirus hemorrhagic disease is a severe and often fatal viral disease in elephants, one of the most significant causes of mortality of juvenile Asian elephants in captivity. The most lethal cases are associated with two types of herpesvirus, EEHV1A and EEHV1B. This case report documents a fatal co-infection of *Clostridium perfringens* type C and elephant endotheliotropic herpesvirus hemorrhagic disease, caused by EEHV4, in an adult female Asian elephant. This report highlights the importance of having both EEHV-HD and clostridial enterotoxemia as potential differential diagnoses in cases of widespread tissue necrosis and internal hemorrhage in elephants, due to their macroscopic similarities, frequent co-occurrence and severity in captive Asian elephant populations.

**Abstract:**

Elephant endotheliotropic herpesvirus hemorrhagic disease (EEHV-HD) is an acute, often fatal, multisystemic hemorrhagic disease and one of the most significant causes of mortality of Asian elephants in captivity. Most fatal cases of EEHV-HD are associated with EEHV1A and EEHV1B in juveniles. This case report describes the clinical and pathological features of a fatal co-infection of *Clostridium perfringens* type C and EEHV-HD, caused by EEHV4, in an adult female Asian elephant. Although fatal clostridial enterotoxemia has been occasionally reported in elephants, this report highlights the importance of having both EEHV-HD and clostridial enterotoxemia as potential differential diagnoses in cases of widespread tissue necrosis and internal hemorrhage in elephants, regardless of the animal age group, due to their macroscopic similarities, frequent co-occurrence and cumulative morbid potential.

## 1. Introduction

Elephant endotheliotropic herpesvirus (EEHV) infection is one of the most significant causes of mortality in captive Asian elephants (*Elephas maximus*) [1]. The clinical disease, caused by EEHV, is referred to as EEHV-hemorrhagic disease (EEHV-HD), in order to distinguish between asymptomatic and symptomatic viremic elephants [2]. EEHV-HD is a major threat for captive Asian elephant population sustainability, particularly in Europe [2,3] and North America [4], but it has also been reported in free-living Asian elephants [5,6,7]. In contrast, there have, as yet, only been a few reports of EEHV infection and EEHV-HD in captive African elephants (*Loxodonta africana*) [5,8,9,10,11].

EEHV, a double-stranded DNA betaherpesvirus (family *Herpesviridae*; subfamily *Betaherpesvirinae*; genus *Proboscivirus*), is nearly ubiquitous in adult elephants [12,13] and the seven EEHV subtypes that have been reported have co-evolved alongside their host elephant species [5]. It has been shown that adult Asian elephants are naturally infected with EEHV1A, EEHV1B, EEHV4 and EEHV5 [13], while African elephants harbor EEHV2, EEHV3, EEHV6 and EEHV7 [5]. The highest incidence of fatal cases in Asian elephants is associated with EEHV1 [5,14], while most of the lethal cases in African elephants have been associated with EEHV3 [8,9]. EEHV-HD caused by a fatal co-infection of EEHV1 and EEHV4 has been reported in a two-year-old Asian elephant in an European zoo.

EEHV-HD was responsible for 57% and 58% of deaths in captive-born Asian elephants in Europe (between 1985 and 2017) [3] and the United States of America (between 1962 and 2007) [5], respectively. Although Asian elephants between one and eight years of age are at higher risk of developing EEHV-HD [3,5], older elephants are also susceptible to EEHV-HD. For instance, there are reports of mortality in a 15-year-old Asian elephant [5] and a 13-year-old African elephant [15], as well as morbidity followed by recovery in an 18-year-old Asian elephant [16]. Although recent evidence suggests that EEHV-HD (caused by EEHV1) is due to primary infection [12,14], recrudescent viremia of a latent EEHV 3/4 infection, secondary to other diseases, such as necrotizing enteritis, has been reported [17].

The severity of clinical signs ranges from subclinical EEHV infection to severe hemorrhagic disease. In juvenile Asian elephants, preclinical EEHV viremia is often characterized by mild to moderate leukopenia and thrombocytopenia, and moderate to severe monocytopenia [10,18,19,20]. Early clinical EEHV viremia frequently starts with non-specific clinical signs, and may include pyrexia, lethargy, changes in sleep patterns, swelling of the temporal gland, changes in defecation, scleral injection, colic, lethargy, changes in food and water intake, lameness and stiffness [2,5]. Affected animals deteriorate rapidly and the clinical disease becomes more severe, with tachycardia, bruising and hemorrhages, cyanosis of the tongue, edema (primarily of the head, trunk, neck and forelimbs), ascites, pericardial fluid (detected via ultrasonography) and bloody diarrhea [2,20,21]. Although recovery has been documented [11,16,18,19,22,23], affected animals often die within seven days of the first clinical signs [16,21], especially if early onset supportive treatment is not provided [16,18,20,24].

The gross pathological features in EEHV-HD are manifestations of acquired thrombocytopenia, disseminated intravascular coagulation and vascular damage from viral-mediated injury to the endothelial cells [2]. The main macroscopic findings of EEHV-HD include pericardial effusion, with extensive petechial to ecchymotic hemorrhage on the surface of the epi- and endocardium and throughout the myocardium, multifocal petechial hemorrhages within the visceral and parietal peritoneal serous membranes, widespread edema (trunk, head, neck, limbs, lung, dependent abdomen, intestinal wall, mesentery) [5,21], hepatomegaly and intestinal hemorrhage and ulceration [25]. Intracranial hemorrhage and blood in cranial cavities may also be observed [26].

Histopathological findings include multifocal areas of hemorrhage, lymphohistiocytic vasculitis, edema and intranuclear eosinophilic inclusion bodies in the endothelial cells of affected tissues [21,27]. Intranuclear eosinophilic inclusion bodies in endothelial cells are not always observed in EEHV4 cases [28]. A recent study has hypothesized that the marked increase in the expression of pro-inflammatory cytokine mRNA could be attributed to the systemic inflammation and disruption of small blood vessels, which is then followed by a disseminated intravascular coagulopathy that enhances hemorrhagic and edematous lesions observed in EEHV-HD [16].

Fatal cases of enteric bacterial infection have been previously reported in elephants. Fatal enterotoxemia, caused by *Clostridium perfringens* type A^β2^, has been reported in an 8-year-old Asian elephant with necrohemorrhagic enteritis and necrotic typhlocolitis [29] and in a 22-year-old African elephant with ulcerative enteritis (linear ulcers) [30]. Toxins A and B expressing *Clostridioides difficile* have been reported in two adult Asian elephants (36- and 46-years-old) with ulcerative enteritis (deep circular ulcers in jejunum and linear ulcers in ileum) and fibrinonecrotic enterocolitis [31]. In some cases, enteric bacterial infection has been associated with concurrent EEHV infection. For instance, *C. perfringens* type B has been reported in a 7-month-old Asian elephant with intestinal oedema and congestion, associated with an EEHV4 infection [32], and in a 3-month-old Asian elephant with necroulcerative gastritis and hemorrhagic enteritis, associated with EEHV1A infection [32]. Similarly, *Salmonella saintpaul* septicemia and ileus has been reported in a 37-year-old female Asian elephant, with clinical evidence of necrotizing enteritis, associated with a recrudescent EEHV3/4 infection [17].

The present case features a fatal co-infection of *C. perfringens* type C and EEHV-HD, caused by EEHV4 in an adult female Asian elephant in the United Kingdom.

## 2. History and Case Presentation

A 20-year and 8-month-old female, approximately five months pregnant (based on copulation dates), captive-born Asian elephant, with a body weight of 3040 kg, was part of a multigenerational family group with three breeding cows (case animal, her mother and daughter) and their three calves at foot. There was also one elderly unrelated female continuously mixed with the family group and a mature bull that was mixed daily with the cow herd. The elephants were managed in protected contact. Diet included hay and straw mix, browse, and pellets (Supplementary Table S1). The case elephant was fully vaccinated with tetanus toxoid (2 mL, unspecified brand in clinical notes) in 2010, in line with the guidelines of the European Association of Zoos and Aquaria (EAZA). In the few weeks preceding the clinical presentation, there were two episodes of social conflict for dominance between the case elephant and her dam, the matriarch, during a period of illness in the latter (deterioration of chronic arthritis), in which the case animal briefly, subtly asserted herself. This conflict was resolved a few weeks before the first clinical signs were observed, when the matriarch improved clinically and the case elephant was, in turn, put back in her place. There were no other husbandry alterations or other obvious external stressors in the weeks prior to the clinical presentation. The last animal imported to the group was the bull in 2012 and the last export was an older cow in 2014.

In September 2018, this adult female Asian elephant presented with an acute onset of lethargy, stretching of the hindlimbs and neck, poor trunk tone and reduced defecation, with normal fecal consistency. A complete physical examination and whole blood sampling were not performed due to the animal being non-compliant with behavior training for veterinary access. A moderate bout of colic was suspected and treatment initiated with spasmolytics (N-butylscopolammonium bromide, Buscopan^®^ Injectable Solution, 20 mg/mL, Boehringer Ingelheim Animal Health, Bracknell, UK, 0.25 mg/kg) and analgesic (butorphanol tartrate, Alvegesic^®^ vet., 10 mg/mL, Dechra Veterinary Products Limited, Shrewsbury, UK, 0.003 mg/kg) delivered by remote intramuscular injection. No improvement was observed, and the animal collapsed and died approximately three hours after initial presentation (two and a half hours after treatment was initiated).

**Figure 1:**
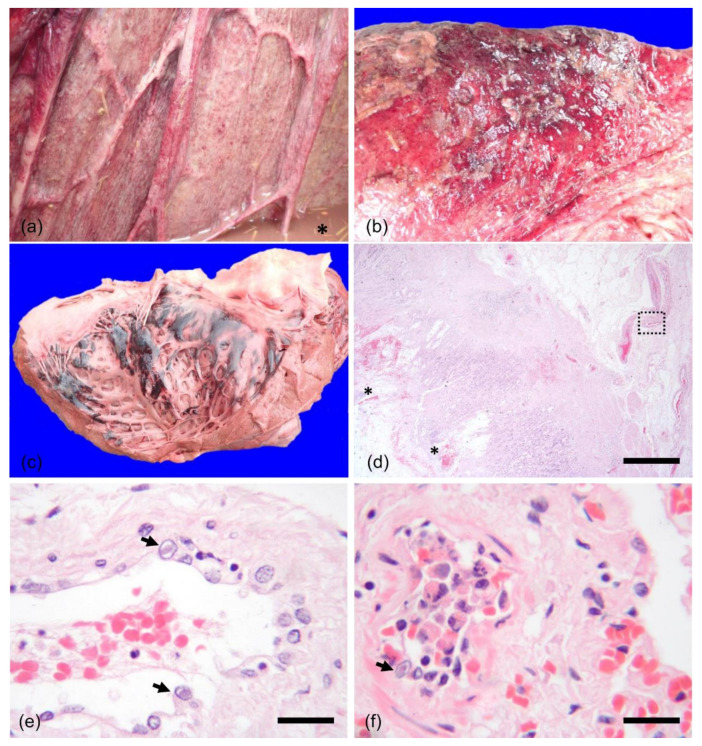
Macroscopic images and photomicrographs of a female adult Asian elephant co-infected with *C. perfringens* type C and EEHV4. (**a**) Proximal colon; myriads pinpoint hemorrhages are visible over the mucosa and fluid content visible at the bottom right corner (asterisk). (**b**) Proximal rectum; large confluent area of mucosal erosion and fibrin deposition. (**c**) Heart, left ventricle; severe multifocal to coalescing subendocardial hemorrhages. Photomicrographs of the large intestine, showing (**d**) area of mucosal erosion with bacteria and hemorrhages (asterisks), also extending to the lamina propria. HE, 4× magnification, bar = 500 µm. Within the edematous submucosa, dotted box highlights the vessel in (**e**), where intranuclear eosinophilic inclusion bodies are visible within enlarged endothelial cells (arrows). HE, 40× magnification, bar = 50 µm. (**f**) Medium sized artery within the pulmonary interstitium with subtotal luminal occlusion by degenerate and necrotic leukocytes and endothelial cells. A single intranuclear inclusion body is evident (arrow). HE, 40× magnification, bar = 50 µm.

The cadaver was kept at room temperature (between 12 and 15 °C) and a post-mortem examination was performed 20 h after death. The animal was in good body condition and there was moderate post-mortem autolysis. The stomach was empty and showed numerous large patches of mucosal hemorrhage and scant luminal turbid fluid. Affecting the entire small intestine and, less severely, the large intestine there was multifocal fibrino-necrotizing and ulcerative enterocolitis, with crater-like mucosal ulcers along the ileum and myriads pinpoint mucosal hemorrhages over the colon (Figure 1a), finally leading to large plaques of mucosal erosion and fibrin deposition over the distal colon and rectum (Figure 1b). Disseminated multifocal to confluent petechial hemorrhages were noted beneath the serosa of the small and large intestines, while various degrees of transmural oedema were visible throughout the small and large intestine. There were hemorrhages and edema of the proximal mesenteric artery, pancreatic hemorrhages and hepatic congestion.

The larynx and, to a lesser extent, the pharynx were reddened and slightly edematous. There was a small volume of red-tinged froth in the larynx, trachea and bronchi. Lungs showed a diffuse dark red discoloration and a wet appearance, consistent with mild pulmonary edema. Numerous coalescing subendocardial hemorrhages were evident along the wall of the left ventricle (Figure 1c).

The spleen was moderately enlarged with a bright red, wet cut surface, and a splenic infarct at its caudal pole. The left prescapular lymph node was markedly enlarged (12 cm maximum length), with a gritty capsule and light-yellow purulent contents. Other cervical lymph nodes were slightly enlarged, with wet and dark red cut surface and scattered cortical light brown to yellow foci. Adrenal glands showed diffusely dark red discoloration of the cortex and, less severely, the medulla (adrenal hemorrhage).

One uterine horn was distended with a thick edematous wall, and contained an amniotic sac of approximately 30 cm, with a fetus inside. Ovaries appeared multi-lobated and hemorrhagic and a large (4 cm diameter) corpus luteum within one of the ovaries was grossly discriminated. The vagina was slightly swollen and contained a cervical mucus plug. Apart from marginal post-mortem changes, the fetus and the placenta showed no gross pathological abnormalities.

Histologically, along the submucosa of the proximal large intestine and distal small intestine, focally over the gastric serosa, disseminated within the interstitium of lung and subendocardial endomysium of the heart, medium caliber arteries and rarely small capillaries exhibited plump endothelium with intranuclear eosinophilic viral inclusion bodies and chromatin marginalization, associated with intravascular fibrin polymerization, granulocytes recruitment and karyorrhexis of sloughed endothelial cells. The intestinal mucosa was often denuded, with disseminated small and coalescing hemorrhages and moderate oedema, which also extended to the submucosa (Figure 1d). Periodic erosions and ulcerations of the mucosa were associated with larger areas of hemorrhage, dense aggregates of necrotic enterocytes and large colonies of cocci and bacilli. In co-localization with areas of intestinal ulceration and hemorrhages (Figure 1d), submucosal and mucosal vessels consistently showed cytopathic changes of the endothelium with intranuclear eosinophilic viral inclusion bodies (Figure 1e), luminal fibrin polymerization and neutrophilic recruitment.

The lungs showed moderate and diffuse congestion with scant alveolar oedema. Within the pulmonary interstitium, intranuclear eosinophilic inclusion bodies could be seen in a few endothelial cells along the intima of medium sized arteries (Figure 1f). Within the right cardiac ventricle, a single endothelial cell of a small caliber vessel presented an intranuclear eosinophilic inclusion body, whereas subendocardial endomysium appeared flooded by extravasated erythrocytes and initial influx of recruited neutrophils.

The left prescapular lymph node showed a central core of eosinophilic amorphous cellular debris and scattered foci of mineralization, surrounded by a thick fibrous capsule and a thin layer of mature lymphoid tissue with scattered foci of necrosis.

Other histological lesions included congestion of the kidney and urinary bladder, and focal chronic suppurative myositis of the superficial left pectoral muscle. No histopathological examination of the placenta and the fetal tissue was performed.

A blood sample collected during post-mortem examination was tested for the presence of EEHV1 (1A and 1B) [33] and EEHV3/4 [34] DNA by quantitative real time PCR (Animal Plant and Health Agency-Weybridge, England, UK). The blood sample was negative for the presence of EEHV1 DNA and was positive for the presence of EEHV3/4 (cycle threshold value of 21.65). The EEHV3/4 PCR amplicon was further resolved by sequencing (Animal Plant and Health Agency-Weybridge, England, UK) and the presence of EEHV4 was confirmed.

Samples of lung, liver, spleen, kidney, small intestine, abdominal fluid and abscess material were submitted for routine bacterial culture on non-selective and selective media including 5% sheep blood agar (BA) and fastidious anaerobe agar (FAA) with 7% defibrinated horse blood for all specimens, in addition to anaerobe BA with neomycin and Brazier selective Agar (for the targeted isolation of *C. difficile*) for the intestinal and abdominal fluid samples (all media from Oxoid, Basinkgstoke, UK) except for FAA obtained from E&O Laboratories, Bonnybridge, UK). Culture was performed aerobically and anaerobically at 37 °C for seven days. Bacterial identification of the isolated organisms was achieved using API^®^ systems (bioMérieux SA, Marcy-l’Etoile, Rhône, France). Significant bacterial growth was only obtained from the small intestine and peritoneal fluid, from which a heavy mixture of coliforms, enterococci and clostridia were cultured. No *Salmonella* spp. or *Shigella* spp. were isolated. Clostridia accounted for the predominant anaerobic growths from both sites, with *C. perfringens* and *C. septicum* isolated from the small intestine, and *C. septicum* and *C. sordellii* isolated from the abdominal effusion. No *C. difficile* was obtained.

Genotypic confirmation of *C. perfringens* and toxinotyping were performed by multiplex PCR assays [35,36]. *C. perfringens* isolated from the small intestine was typed to toxin type C, carrying the α and β -toxin genes *cpa* and *cpb*. The isolate tested negative for *cpb2*, *etx*, *iA* and *cpe* toxin genes. Molecular typing of the *C. septicum* and *C. sordellii* isolates were not pursued on this occasion. Antimicrobial susceptibility testing of clostridial isolates by the disc diffusion method showed broad susceptibility of *C. perfringens* to the antibiotics tested (penicillin, clindamycin and metronidazole).

## 3. Discussion

The combined findings of the post-mortem examination, histopathology, microbiology and molecular analysis showed that this elephant was co-infected with EEHV4 and *C. perfringens* type C (with the potential contribution of *C. septicum*).

EEHV-HD was first detected in this herd in 2009. Since then, the herd has lost a total of seven calves, all of them to EEHV1 (five calves died before the case elephant, and two calves died after that). Since the death of the case animal, a calf at this collection has been successfully treated for EEHV1A-related hemorrhagic disease [24]. The EEHV status of this herd is closely monitored. Until the death of the case elephant, adults in the herd were tested weekly for the presence of EEHV1 DNA shedding via oral/trunk swabs, while calves (all under three years of age) were tested weekly (or twice weekly) for the presence of EEHV1 DNA in blood. EEHV1 shedding and DNAemia are intermittently detected in the herd, primarily from calves. Since 2018, after EEHV4 was diagnosed in the case elephant, oral/trunk swabs from adults and blood from calves are also tested for the presence of EEHV3/4 DNA. The herd has not had any other positive results for the presence of EEHV3/4 DNA in any of the samples tested. Moreover, there is no recent history of importation into the herd (the last one was six years earlier, in 2012, with the importation of the bull). Unfortunately, data on the serological EEHV status of the herd were not available. This data could have allowed us to infer whether primary infection or recrudescence of latent infection occurred. However, taking together the absence of subsequent EEHV3/4 shedding/DNAemia in the herd and the absence of a recent introduction of new animals into the herd, we speculate that, in the present case, a recrudescence of a latent EEHV4 infection is more likely than a primary infection.

While EEHV viremia has been associated with the reactivation of an EEHV3/4 latent infection [17], some authors suggest that primary infection (and not the reactivation of a latent infection) is an underlying factor for EEHV1-related hemorrhagic disease in Asian elephants [12,14] and EEHV3-related hemorrhagic disease in African elephants [9], which may suggest a variation in pathogenicity amongst viral strains.

We are unable to establish whether this was a primary case of EEHV4 infection with secondary clostridial enterotoxemia, or whether this was a primary clostridial enterotoxemia with secondary EEHV4 infection. However, the very close spatial association between the cytopathic effects of the virus on endothelial cells and the hemorrhagic and ulcerative lesions observed histologically suggests that the EEHV infection played a relevant role in the current presentation. Furthermore, the observation of viral inclusions in distant organs (e.g., lung) outside the gastrointestinal system and the high level of EEHV4 DNAemia supports the existence of generalized viral replication with viraemia in this animal. Co-infection of enteric bacteria and EEHV has been previously reported in Asian elephants, and includes *C. perfringens* type B and EEHV [1A and 4] in calves [32], and *S. saintpaul* and EEHV3/4 in an adult female [17]. In the present case, we speculate that the fibrino-necrotizing and ulcerative enterocolitis were due to the effects of toxin-producing *C. perfringens* type C on the gastrointestinal tract, which then exacerbated the clinical response to the EEHV4 infection. The widespread petechial and ecchymotic hemorrhages observed on the endocardium and myocardium, abdominal serosal membranes, and root of the mesentery are compatible with EEHV-HD [2]. Based on the above results, we conclude that this animal had an active EEHV4 infection, and that the associated hemorrhagic disease significantly contributed to its death.

On the other hand, viral-induced immunosuppression can predispose individuals to bacterial infections. For instance, *C. perfringens* infection has been linked with fatal EEHV-HD in Asian elephant calves [32], as well as in European badgers (*Meles meles*) with genital tract gammaherpesvirus (*Mustelid gammaherpesvirus 1*) [37], in dairy cows with ulcerative endometritis caused by *Bovine herpesvirus 4* [38] and in other captive artiodactyls with gammaherpesvirus infections [39]. Similarly, lymphohistiocytic colitis caused by *C. piliforme* has been reported in a kitten in association with rhinitis, tracheitis and bronchointerstitial pneumonia, caused by *Felid herpesvirus 1* [40].

Other risk factors for *C. perfringens* enteritis and enterocolitis include coprophagia, often observed in young elephants [32], and diet changes (e.g., high protein or carbohydrate) [41,42,43]. Co-occurrences, rather than co-infections, can ultimately be a consequence of dysbiosis [44]. Determining the significance of clostridia requires a combination of tests, including isolation and identification of the bacteria, characterization of its toxigenic potential and the presence of toxins within the tissues and fluids collected during post mortem examination [45], as the detection of the genes encoding for the production of toxins alone does not prove that toxins are present at the site of the lesions.

Although clostridia, including *C. perfringens*, have previously been associated with enterocolitis in elephants, they are rarely encountered in the intestinal tract of healthy elephants [30,46]. Therefore, we believe that *C. perfringens* type C, isolated in the present case, has played an important pathogenic role. The clinical relevance and pathogenicity of *C. septicum* and *C. sordellii* in this study remain unknown, as no further molecular characterization was performed on these isolates. *C. septicum* is regarded as an important post-mortem invader [47], and is responsible for myonecrosis in humans and braxy and malignant edema (gas gangrene) in ruminants, as well as clostridial dermatitis in poultry [48]. Both *C. septicum* and *C. sordellii* have been sporadically implicated in individual cases of enteric diseases in animals and/or humans [49,50,51].

Physiological [13] and behavioral stressors [52] within the herd have been suggested as a possible cause of the increased frequency and quantity of EEHV viral shedding. Increased viral shedding in pregnant Asian elephants has been reported [33,53], but it is not consistently observed [52]. In humans, relative immunosuppression and enhanced immune tolerance is a well-described phenomenon, necessary for the survival of the foetus [54]; thus, a similar effect could be postulated in other mammals. Taken together, the early pregancy in this animal, the recent temporary herd dynamic shift (when this animal briefly asserted herself while her dam, the matriarch, showed some deterioration as a result of chronic arthritis), the absence of a recent animal import into the herd and the absence of EEHV4 shedding/DNAemia detection since the death of the case elephant, it is plausible to speculate that immunosuppression could have played a role by facilitating a recrudescence of a latent EEHV4 infection.

Although no macroscopic lesions were observed on the placenta or the fetus, histopathology was not performed. Therefore, histological lesions on these tissues cannot be ruled out. A comprehensive analysis of the placenta and the fetus of pregnant elephants that suffer abortions or die of EEHV-HD are encouraged in order to assess the effect of this virus in elephant pregnancy.

Macroscopic gastrointestinal lesions of EEHV-HD may resemble other diseases [55]. For instance, gross findings of EEHV infection include diffusely scattered petechiae within visceral and parietal peritoneum, large intestinal ulcers, and hemorrhages throughout the stomach, small intestine and large intestine [25]. Similar gross lesions caused by other gastroenteric pathogens include necroulcerative gastritis (*C. perfringens* type B) [32]; hemorrhagic (*C. perfringens* type B) [32], necrotizing (*S. saintpaul*) [17] and necrohemorrhagic (*C. perfringens* type A^β2^) [29] enteritis; ulcerative enteritis (due to *C. perfringens* type A^β2^ [30] and *C. difficile* [31]); fibrinonecrotic (*C. difficile*) [31] and ulcerative and hemorrhagic (*S. blockey*) [56] enterocolitis; necrotic typhlocolitis (*C. perfringens* type A^β2^) [29]. Similarly, cardiopulmonary lesions caused by EEHV are similar to those observed in other bacterial infections. For example, atrial and ventricular endo- and epicardial hemorrhage, hydropericardium, pleural hemorrhage, and pulmonary congestion and edema have been reported in an adult African elephant infected with *Citrobacter freundii* [57]. Widespread hemorrhage can also be observed in vitamin E deficiency [55]. Therefore, apart from EEHV infection, differential diagnosis for widespread necrosis and hemorrhage in elephants should include other infectious and non-infectious diseases.

## 4. Conclusions

This case report describes the clinical and pathological features of a fatal co-infection of EEHV4 and *C. perfringens* type C in an adult Asian elephant. Although we were unable to establish whether this was a primary case of EEHV infection with secondary clostridial enterotoxemia or vice versa, we speculate that the fibrino-necrotizing and ulcerative enterocolitis were due to toxin-producing *C. perfringens* type C effects on the gastrointestinal tract, which then exacerbated the clinical response to EEHV4 infection. We also speculate that immunosuppression could have played a role by facilitating a recrudescence of a latent EEHV4 infection.

Based on the gross findings compatible with EEHV-HD, the active EEHV4 viral replication observed histologically, and the high levels of EEHV4 DNAemia, we conclude that this animal died of a hemorrhagic disease caused by EEHV4. This is the first report of a fatal co-infection of EEHV4 and *C. perfringens* in an adult Asian elephant.

The present case highlights the importance of including EEHV and clostridial infections as potential differential diagnoses in cases of widespread necrosis and hemorrhage in elephants. The intestinal pathology of EEHV is only sketchily described, and its role in generating ulcerative and necrohemorrhagic gastroenteritis and/or colitis warrants further investigation, as does the potential synergy between enteric flora in contributing to viral morbidity and mortality, particularly in adult elephants.

## Data Availability

Not applicable.

## Supplementary Materials

The following supporting information can be downloaded at: https://www.mdpi.com/article/10.3390/ani12030349/s1, Table S1: Diet offered daily to a female adult Asian elephant in 2018.
