# Supplementary material for: Elephant Endotheliotropic Herpesvirus 4 and Clostridium perfringens Type C Fatal Co-Infection in an Adult Asian Elephant (Elephas maximus)
Source: Animals (Basel). 2022 Jan 31;12(3):349. doi: 10.3390/ani12030349 (PMC8833544; doi:10.3390/ani12030349)
Supplement: Supplementary file 1 [file animals-12-00349-s001.zip › animals-1443554-supplementary.pdf]

**Table S1.** Diet offered daily to a female adult Asian elephant in 2018.

| Food item * | Approximate quantity |
|---|---|
| Grass hay | 1.5 bales |
| Straw | 4 kg |
| Browse (thin, leafy branches) | 35 kg |
| Elephant cubes (Dodson and Horrell) | 2-4 kg |
| High fibre nuts (HJ Lea Oakes) | 1.5 kg |
| Low – Pro Elephant Rolls (HJ Lea Oakes) | 2 kg |
| Dog biscuit (Pointer) | 1.5-3 kg |
| Carrots | 4 kg |
| Apples and banana | 4 kg |

* Daily rations were continuously adjusted by elephant keepers in response to close monitoring of body weight and condition of the animals. Hay was offered throughout the day from feeders in the paddock and hay nets in the pens; browse was distributed around the paddock in the morning and in the pens in the afternoon; pellets were fed using randomised feeders, enrichment balls or scattered; dog biscuits were generally used for behavior training; produce was used for scatter feeding and/or burying in the paddock to promote foraging.
